# Voting behavior during FDA Medical Device Advisory Committee panel meetings

**DOI:** 10.1371/journal.pone.0267134

**Published:** 2022-06-24

**Authors:** Amanda Maisel-Campbell, Daniel I. Schlessinger, Arianna F. Yanes, Emir Veledar, Kelly A. Reynolds, Sarah A. Ibrahim, Bianca Y. Kang, Noor Anvery, Emily Poon, Murad Alam

**Affiliations:** 1 Department of Dermatology, Feinberg School of Medicine, Northwestern University, Chicago, IL, United States of America; 2 Department of Dermatology, Columbia University Medical Center, New York, NY, United States of America; 3 Division of Dermatology, Washington University School of Medicine, St Louis, MO, United States of America; 4 Department of Dermatology, University of Pennsylvania, Philadelphia, PA, United States of America; 5 Emory University School of Medicine, Atlanta, GA, United States of America; 6 Baptist Health South Florida, Miami, FL, United States of America; 7 University of Cincinnati College of Medicine, Cincinnati, OH, United States of America; 8 Department of Otolaryngology, Feinberg School of Medicine, Northwestern University, Chicago, IL, United States of America; 9 Department of Surgery, Feinberg School of Medicine, Northwestern University, Chicago, IL, United States of America; University of Hong Kong, HONG KONG

## Abstract

**Objectives:**

During premarket review, the US Food and Drug Administration may ask its Medical Device Advisory Committee (MDAC) Panels to assess the safety and effectiveness of medical devices being considered for approval. The objective of this study is to assess the relationship, if any, between individual votes and Panel recommendations and: (1) the composition of Panels, specifically the expertise and demographic features of individual members; or (2) Panel members’ propensity to speak during Panel deliberations.

**Methods:**

This was a retrospective cohort study of routinely collected data from voting members of MDAC panels convened between January 2011 to June 2016 to consider premarket approval. Data sources were verbatim transcripts available publicly from the FDA. Number of words spoken, directionality of votes on device approval, profession, and demographics were collected.

**Results:**

658,954 words spoken by 536 members during 49 meetings of 11 Panels were analyzed. Based on multivariate analysis, biostatisticians spoke more (+373 words; *P* = 0.0002), and women (-187 words; *P* = 0.0184) and other non-physician voting members less (-213 words; *P* = 0.0306), than physicians. Speaking more was associated with abstaining (*P* = 0.0179), and with voting against the majority (*P* = 0.0153). Non-physician, non-biostatistician members (*P* = 0.0109), and those having attended more meetings as a voting member (*P* = 0.0249) were more likely to vote against approval. In bivariable analysis, unanimous Panels had a greater proportion of biostatisticians (mean 0.1580; 95% CI 0.1237–0.1923) than non-unanimous Panels (0.1107; 95% CI 0.0912–0.1301; p = 0.0201).

**Conclusions:**

Panelists likely to vote against the majority include non-physician, non-biostatisticians; experienced Panelists; and more talkative members. The increased presence of biostatisticians on Panels leads to greater voting consensus. Having a diversity of opinions on Panels, including in sufficient numbers those members likely to dissent from majority views, may help ensure that a diversity of opinions are aired before decision-making.

## Introduction

The United State Food and Drug Administration (FDA) is responsible for regulating, reviewing, and approving medical drugs, devices, and cellular therapies for routine patient use. To be approved, novel devices must be determined to be safe and effective. While FDA has clear rules for such approvals, and copious preclinical and clinical evidence provided by companies seeking approvals is sifted carefully by highly experienced FDA staff, FDA also engages independent experts to help decide whether specific products should be approved.

Specifically, during premarket review, FDA’s Center for Devices and Radiological Health (CDRH) may ask its Medical Device Advisory Committee (MDAC) Panels, comprised of independent experts, to assess the safety and effectiveness of medical devices being considered for approval. Although Panel recommendations are not binding, they greatly influence subsequent decisions by the FDA Commissioner. One study found that FDA approved nearly 90% of original new drug and biologics license applications supported by its Advisory Committee Panels [1].

Given the high degree of influence of Advisory Panels on approvals, it is important to understand how such Panels operate and how they arrive at their recommendations. Beyond the formal process of evidence review and question and answer, Panels are impacted by group dynamics that are affected by the specific members and experts who are included, their demographic features, how much each speaks, and how they interact. While the impact of group dynamics in FDA Panels is relatively poorly understood, further exploration of these issues may help uncover weaknesses that can be corrected or improved. The end result may then be a better Panel process, which is more resistant to bias, and more likely to produce good decisions. While the current FDA process for premarket approval is a gold standard worldwide, emulated by regulatory authorities in other countries, there may be an opportunity to make it even better.

The four principal types of participants in typical MDAC Panel meetings are the committee members, the chair, the FDA professional staff, and the sponsor of an application. Committee members (henceforth referred to as Panel members, or Panelists) are nominated to the MDAC based on the relevance of their area of expertise and the extent to which they have only limited conflicts of interest which would not preclude their serving. Most committee members have other principal employment and are brought on as special government employees, with their assignment to specific Panels based on their suitability to evaluate the devices or questions under review. Members can be appointed to a particular Panel for several years, or they can be assigned on an *ad hoc* basis as their expertise is required (e.g., Temporary Panel Members). CDRH ensures that each Panel includes at least 2 voting members with clinically relevant expertise and one voting member who is knowledgeable about the technology of the device. Most Panels have at least one biostatistician, who may for instance, be a faculty member at an academic medical center. At the discretion of FDA staff, more than one biostatistician may be assigned to a particular Panel, presumably when there is need for more complex data review, although the reasons are not typically conveyed. Panels are comprised of physicians, non-physician experts, and biostatisticians, each recruited based on their expertise in the field.

Non-physician experts may be selected from a very large list of possible professions, depending on the specific Panel and the particular device under consideration [2]. For the Panels assessed in this paper, non-physician experts included epidemiologists, bioethicists, geneticists, podiatrists, pharmacists, optometrists, electrophysiologists, engineers, other scientists, and regulatory experts.

Meetings are typically structured with presentations by FDA staff and sponsors first, followed by committee question and answer (Q&A) sessions directed to the FDA and product sponsors, and finally an open public hearing (OPH) Q&A session with other interested parties. After a lengthy discussion, including answering questions posed by FDA to the Panel, committee members cast formal votes on questions related to the approvability of a product, including evaluation of post-market safety data and pre-market risk-benefit profile. The discussion and the votes ultimately help to inform the agency’s final decisions [3, 4].

Several endogenous and exogenous factors that may be associated with Panel recommendations have recently been studied [5–7]. A study conducted by Lurie et al. found that financial ties were weakly associated with votes for approval [6]. Another study reported that seating location–which may determine speaking and voting order–significantly impacted voting behavior [7].

Other factors that may possibly be associated with Panel outcomes include the demographic features and areas of expertise of Panel members, as well as the extent to which individual Panel members speak and debate during Panel meetings. To our knowledge, the association of these factors with the votes of individual members and overall Panel recommendations has not previously been studied. The purpose of this study was to assess the relationship, if any, between votes and Panel recommendations and either: (1) the composition of MDAC Panels, specifically the expertise and demographic features of individual members; or (2) Panel members’ propensity to speak during Panel deliberations. We hypothesized that certain voter characteristics may influence voting behaviors. Specifically, we expected that those who were less likely to vote to recommend approval and more likely to dissent from the majority vote may be more experienced (as measured by the number of Panel meetings attended); non-temporary voting members; more talkative and certain in their spoken opinions; men; and physicians.

## Methods

Data sources were transcripts of US FDA CRDH MDAC Panel meetings convened between January 2011 and June 2016 to consider premarket approval of medical devices [8]. Transcripts of Panel meetings convened for other purposes, such as review of current knowledge or classification or reclassification of devices, were excluded. Only voting members of CDRH MDAC participating in Panel meetings occurring in the designated period were considered in analyses (nonvoting members such as Panel chairs, who only function as tie-breakers, and industry representatives were excluded).

The following variables were collected for each Panel meeting: Panel name; meeting date; names of voting members (gender was deduced from members’ name-for cases in which the name was not commonly associated with a specific gender, we searched for identifying pronouns and photographs of the member on academic or personal websites); member profession; member opinion regarding device safety and efficacy; final member vote regarding device approval recommendation; number of words spoken by each voting member during each major meeting segment (e.g., FDA question and answer [Q&A] session, sponsor Q&A session, Panel deliberations (includes deliberations regarding official questions posed to panelists by the FDA), and open public hearing [OPH] Q&A session); and the number of meetings attended by each member (among the meetings included in our sample- several Panel members attended multiple meetings and thus were represented several times in our sample of voting members. The variable “number of meetings attended” was coded sequentially, in chronological order, for each Panel member). Additionally, whether or not the Panel officially recommended device approval was determined from individual member votes.

### Statistical analysis

Student’s two-tailed t-tests and Pearson chi-square tests were used to compare continuous and categorical variables, respectively, between subgroups. Variables that had a *p-*value <0.10 were included in multivariable models. A multivariable linear regression model was used to evaluate member characteristics associated with changed in voter talkativeness (expressed as beta coefficients). Odds ratios (ORs) with 95% Wald confidence intervals (CIs) were estimated using a logistic regression models to assess for member characteristics associated with dissenting voters (defined as voting members who voted in disagreement with the majority Panel vote regarding final approval recommendation) and with final member vote regarding device approval recommendation. P< 0.05 was considered statistically significant. Statistical analyses were performed using SAS Studio software (version 3.71; SAS Institute Inc.)

## Results

### Panel characteristics

Forty-nine meetings of 11 Panels were included. The mean number of meetings per Panel was 4.45 (95% confidence interval [CI], 0.82–8.09). The number of voting members in attendance ranged from 6 to 18 (mean, 10.7; 95% CI, 10.04–11.43).

The majority vote in 43 meetings (88%) was in favor of device approval. In all meetings in which the majority was *not* in favor of recommending device approval, the majority of the voting members felt that there was neither reasonable assurance of device safety nor effectiveness. Only one meeting (2.0%), the Circulatory Systems Panel meeting on October 8, 2013, resulted in a tie vote (3 for, 3 against), necessitating a tie-breaking vote by the Panel chair, who voted in favor of approval. This vote was excluded from analysis per our eligibility criteria.

### Voting member characteristics

Characteristics of eligible voters are shown in **Table 1.** In 5 cases voting members were absent during voting, and so 521 of 526 eligible votes were actually cast, 440 by men (84%): 361 (69.3%) in favor of device approval, 123 (23.6%) not in favor, and 37 (7.1%) abstentions.

**Table 1:** Characteristics of voting members (N = 526).

Characteristic	No. (%)
**Gender**	
**Female**	116 (22)
**Male**	410 (78)
**Member Profession**	
**Physician**	394 (75)
**Biostatistician**	63 (12)
**Other Non-Physician Expert**	69 (13)
**Status**	
**Temporary Voting Member**	395 (75.1)
**Voting Member**	131 (24.9)
**Number of Meetings Attended, Per Voter** ^**a**^	Mean, 2.03; 95% CI, 1.78–2.29
**Total Words Spoken** ^**b**^ **, Per Voter**	Mean 1252.8; 95%CI, 1187.9–1317.6

^a^ Number of meetings attended as a voting member between January 01, 2011-June 01, 2016

^b^ No. of Words Spoken During All Major Meeting Segments

### Voter talkativeness

**Fig 1:**
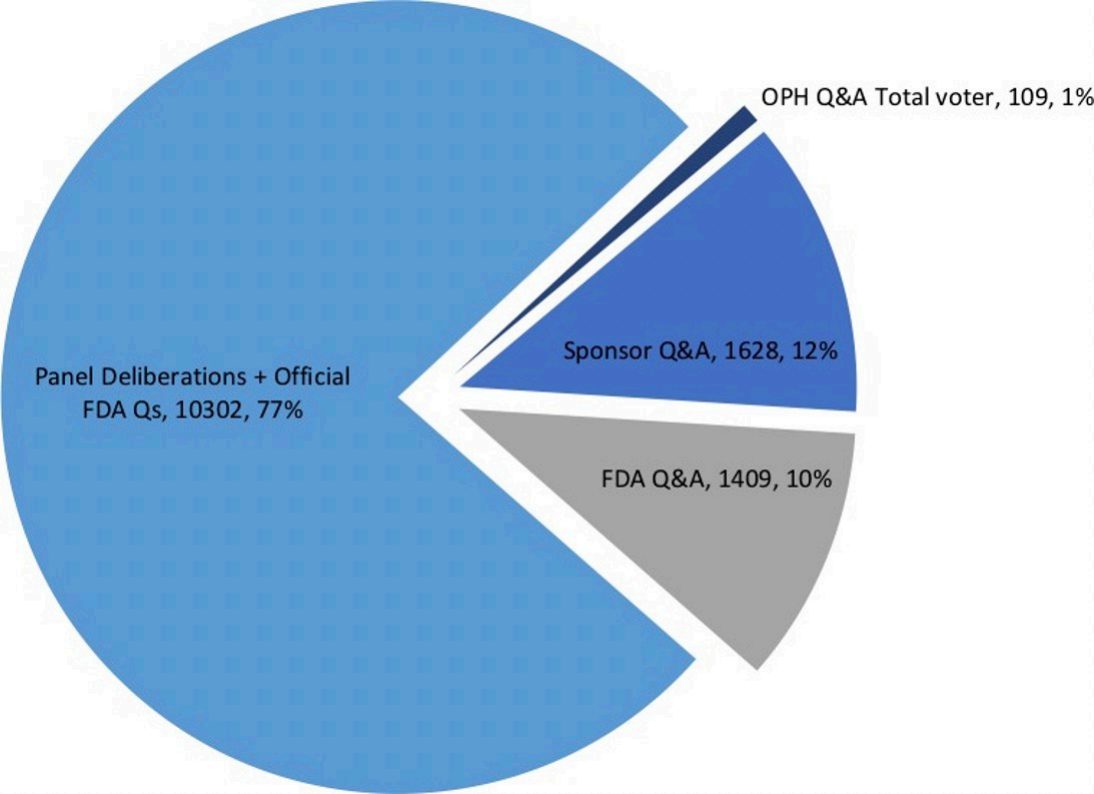
Average number of words spoken by all voting members during major meeting segments, per meeting.

The 658,954 words spoken by voting members during the major segments of the 49 meetings were analyzed. Most words (77%) were spoken late in the meeting during the Panel deliberations while the fewest were during OPH Q&A sessions (1%; **Fig 1**). Of note, voters only spoke during OPH Q&A sessions in 10 meetings (20.4%), during all of which a majority voted for approval. In 2 of these 10 meetings, voting members directed questions to patient speakers. In the remainder of cases, questions were addressed to physician speakers or professional society representatives, typically to request clarification of conflicts of interests or to better understand speaker experience with device usage and adverse effects.

Bivariable analyses showed a significant association between voter talkativeness and member profession and gender (**Table 2**). A multivariable model of voter talkativeness (**Table 2**) found being a biostatistician was independently associated with speaking significantly more (+373 words; *P* = 0.0002), and any other non-physician voting member with speaking less (-213 words; *P* = 0.0306), than physician members. Female gender was also independently and significantly associated with speaking less (-187 words; *P* = 0.0184).

**Table 2:** Relationship between voter characteristics and talkativeness^a^ in the univariable and multivariable linear regression analyses.

		Bivariable		Multivariable	
Characteristic	No. (%)	β Coefficient (95% CI)	*P* Value	β Coefficient (95% CI)	*P* Value
**Member Status**			0.7124		
Temporary Voting Member	395 (75)	28.2 (-121.9 to 178.2)		-	
Voting Member	131 (24)	1 [Reference]		-	
**Member Profession**					
Biostatistician	63 (12)	360.0 (162.2 to 557.9)	0.0004	373.3 (176.1 to 570.6)	0.0002
Other^b^	394 (75)	-254.3 (-444.6 to -64.0)	0.0089	-212.6 (-405.2 to -20.0)	0.0306
Physician	69 (13)	1 [Reference]		1 [Reference]	
**Member Gender**					
Female	116 (22)	-208.3 (-363.8 to -52.9)	0.0087	-187.0 (-342.3 to -31.7)	0.0184
Male	410 (78)	1 [Reference]			
**Number of Meetings Attended** ^**c**^ **, Per 1 Meeting**		-1.7 (-27.6 to 24.1)	0.8972		

Multivariable model R^2^ (adjusted) = 0.0465; *P*<0.0001

Abbreviations: SD, standard deviation

^a^ Voter talkativeness defined as the number of words spoken by the voter during all major meeting segments (including FDA Q&A, Sponsor Q&A, open public hearing Q&A, and Panel deliberations)

^b^ Other non-physician, non-biostatistician expert voting member (e.g. engineer, electrophysiologist, etc.)

^c^ Number of meetings attended as a voting member between January 01, 2011-June 01, 2016 that were included in our sample

### Final member vote

Bivariable analyses showed a significant relationship between final member vote (final member vote defined as the member’s vote at the conclusion of the Panel deliberations regarding whether or not to recommend device approval) and: (a) member profession; (b) voter certainty (certain voters were defined as those that expressed confidence, or lack thereof, in both the safety and effectiveness of the device; uncertain voters expressed confidence that the device was safe, but not effective, or vice versa); (c) the number of meetings attended by the member; and (d) voter talkativeness. The association between final member vote and voter talkativeness was significant in all major segments of the meeting. A multinomial logistic regression model of final member vote in this sample is shown in **Table 3**. Speaking more during major meeting segments was significantly and independently associated with voting to abstain (*P* = 0.0179). Compared to certain voters, the odds of uncertain voters voting to abstain or voting *not* in favor of approval were nearly 6 and 12 times higher, respectively, than voting in favor of approval (*P* <0.0001 for both associations). Other factors associated with voting *not* in favor of recommending approval were member profession (the odds of non-physician, non-biostatistician voting members being nearly 2.5 times that of physician voters; *P* = 0.0109) and having attended more meetings as a voting member (*P* = 0.0249).

**Table 3:** Bivariable and multivariable multinomial logistic regression for factors associated with voting to abstain or voting “not in favor” during the final device approval recommendation vote.

	Bivariable		Multivariable	
Voter Characteristics	Members Who Voted to Abstain (n = 37)	Members Who Voted to Not Approve (n = 123)	Members Who Voted to Abstain (n = 37)	Members Who Voted to Not Approve (n = 123)
	Odds Ratio (95% CI)		Odds Ratio (95% CI)	
**No. of Meetings Attended** ^**a**^ **, Per 1 Meeting**	0.960 (0.815–1.131)	1.122 (1.042–1.209)^b^	0.932 (0.778–1.115)	1.104 (1.013–1.204)^b^
**Number of Words Spoken** ^**c**^ **:**				
Entire Meeting^d^	1.061 (1.022–1.102)^e^	1.026 (0.999–1.054)	1.050 (1.008–1.093)^b^	1.023 (0.990–1.057)
Sponsor Q&A	0.958 (0.772–1.189)	0.988 (0.872–1.119)		
FDA Q&A	1.010 (0.831–1.229)	0.966 (0.852–1.095)		
Panel Deliberations + Official FDA Qs	1.070 (1.029–1.113)^f^	1.032 (1.003–1.062)^b^		
Open Public Hearing Q&As	0.881 (0.349–2.224)	1.178 (0.765–1.813)		
**Member Gender**				
Female	0.954 (0.420–2.167)	0.927 (0.563–1.525)		
Male	1 [Reference]			
**Member Profession**				
Biostatistician	2.712 (1.178–6.242)^b^	1.741 (0.939–3.228)	2.424 (0.991–5.93)	1.568 (0.760–3.239)
Other^a^	0.505 (0.116–2.202)	1.943 (1.113–3.392)^b^	0.654 (0.146–2.928)	2.360 (1.219–4.569)^b^
Physician	1 [Reference]		1 [Reference]	
**Member Status**				
Temporary Voting Member	0.844 (0.402–1.774)	1.400 (0.851–2.303)		
Voting Member	1 [Reference]			
**Certainty of Votes** ^**b**^				
Uncertain Voter	6.286 (3.058–12.924)^f^	11.958 (7.374–19.391)^f^	6.022 (2.885–12.571)^f^	11.979 (7.294–19.673)^f^
Certain Voter	Reference		1 [Reference]	

Reference group = Members who Voted to Approve (n = 361)

Abbreviations: OR, odds ratio; CI, confidence interval

^a^ Number of meetings attended as a voting member between January 01, 2011 and June 01, 2016. Meetings counted sequentially in chronological order.

^b^ p<0.05

^c^ Per voter, per 100 words

^d^ Total words spoken during major segments of the meeting (Sponsor Q&A, FDA Q&A, open public hearing Q&A, and Panel deliberations)

^e^ p<0.01

^f^ p<0.001

### Characteristics of uncertain voters

Analysis of uncertain voters found that 55.9% (76 of 136) voted *not* in favor of approval (vs 12.2% [47 of 385] of certain voters), 31.6% voted in favor of approval (vs 82.6% of certain voters), and 12.5% voted to abstain (vs 5.2% of certain voters). Of the 43 cases in which uncertain voters ultimately recommended approval, 67.4% (29 of 43) expressed confidence in the safety of the device but were unsure of its effectiveness while 30.2% expressed confidence in device effectiveness but were unsure of its safety. Additionally, there was one unusual case in which a male, non-physician, temporary voting member who had attended no prior Panel meetings abstained from commenting with regard to device safety and expressed a lack of confidence in its effectiveness, but ultimately voted to recommend device approval (which was in agreement with the Panel majority).

### Characteristics of voters not voting with the majority

Not voting with the majority (i.e., abstaining or dissenting) was significantly associated with voter uncertainty and voter talkativeness in both bivariable and multivariable analyses, as shown in **Table 4**. In the multivariable model, the odds of placing a vote in disagreement with the majority was 7 times higher among uncertain voters compared to certain voters (OR 7.098; 95%CI 4.466–11.283; *P*<0.0001). Voting not with majority was also independently and significantly associated with increased talkativeness during major segments of the meeting (*P* = 0.0153).

**Table 4:** Univariable and multivariable analysis of characteristics associated with voters not voting with the majority^a^.

	Bivariable		Multivariable	
	Odds Ratio (95%CI)	*P* Value	Odds Ratio (95%CI)	*P* Value
**Number of Words Spoken** ^**b**^ **:**				
Entire Meeting^c^	1.040 (1.014–1.067)	0.0028	1.038 (1.007–1.069)	0.0153
Sponsor Q&A	1.011 (0.893–1.145)	0.8612		
FDA Q&A	1.027 (0.911–1.159)	0.6624		
Panel Deliberations + Official FDA Qs	1.042 (1.014–1.071)	0.0032		
Open Public Hearing Q&As	1.425 (0.942–2.155)	0.0932		
**Number of Meetings Attended**^**d**^ **(Per 1 Meeting)**	1.069 (0.991–1.154)	0.0828	1.044 (0.957–1.138)	0.3343
**Panel Status**		0.4152		
Temporary Voting Member	0.824 (0.516–1.314)	0.4156		
Voting Member	1 [Reference]			
**Gender**		0.9668		
Female	0.989 (0.599–1.634)			
Male	1 [Reference]			
**Member Profession**		0.0821		
Biostatistician	1.778 (0.982–3.220)		1.436 (0.727–2.839)	0.2976
Other Non-Physician^e^	1.565 (0.872–2.808)		1.818 (0.941–3.513)	0.0751
Physician	1 [Reference]		1 [Reference]	
**Certainty of Votes** ^**f**^				
Uncertain Voter	7.369 (4.671–11.626)	<0.0001	7.098 (4.466–11.283)	<0.0001
Certain Voter	1 [Reference]		1 [Reference]	

Abbreviations: CI, confidence interval

^a^ Regarding the final vote to recommend or not recommend device approval. Voters not voting with the majority were defined as those who voted to abstain or those who voted not in favor when the Panel majority disposition was in favor of device approval or voters who voted in favor of device approval or voted to abstain when the Panel majority disposition was *not* in favor of device approval.

^b^ Per voter, per 100 words

^c^ Total words spoken during major segments of the meeting (Sponsor Q&A, FDA Q&A, open public hearing Q&A, and Panel deliberations)

^d^ Number of meetings attended as a voting member between January 01, 2011 and June 01, 2016. Meetings counted sequentially in chronological order.

^e^ Other non-physician, non-biostatistician expert (e.g. engineer, electrophysiologist, etc.)

^f^ Voter certainty defined as certainty with regard to confidence in the safety and effectiveness of the device. Voters who expressed confidence that the device was safe, but not effective, or vice versa, were considered uncertain voters whereas those who expressed confidence, or lack thereof, in both the safety and effectiveness of the device were considered certain voters

### Characteristics of unanimous votes

Based on bivariable analyses, unanimous Panels had a significantly greater proportion of biostatistician voting members, on average, (mean 0.1580; 95% CI 0.1237–0.1923) compared to Panels in which the final approval vote was not unanimous (mean 0.1107; 95% CI 0.0912–0.1301; p = 0.0201).

### Summary results

Voting against recommending approval at the conclusion of MDAC Panel meetings was found to be associated with greater voter experience, uncertainty in voter opinions, and voter profession, with those who were neither physicians nor biostatisticians more likely to vote against approval. Abstaining during the final vote was associated with voter talkativeness. If abstaining or voting against were collectively classified as not voting with the majority, then talkative voters and uncertain voters were more likely to so vote. Biostatisticians spoke more than physicians, and non-physicians spoke less than physicians. Women spoke less than men.

## Discussion

Some of our hypotheses were borne out, as more experienced members and more talkative members tended to vote against approvals. Contrary to our expectations, those more certain in their spoken opinions were less likely to abstain, vote against approval, or dissent with the majority. Similarly, while profession impacted voting behavior, it was non-physicians rather than physicians who were more likely to vote not to approve. Although the results for temporary voting members did not rise to significance, such members were, surprisingly, nominally less likely to abstain and more likely to vote against approval than non-temporary members.

In interpreting the “number of works spoken” as a measure of “talkativeness,” this paper was consistent with relevant literature. Word count is often used as a primary metric for talkativeness [9]. A separate question was whether some Panelists were garrulous but not imparting much novel information. Interestingly, the political science literature does indicate that talkativeness, as measured by total speaking time irrespective of the semantic content, has been shown to influence group members’ perceptions of who is contributing most to the conversation, and who is a leader within small groups [10]. Thus, Panelists who are very talkative, even when they are repetitive and uninformative rather than eloquent or thoughtful, may be able to sway the opinions of the Panel to some extent.

The result that speaking more was associated with abstaining or with voting against approval was unexpected and yet interesting. A possible interpretation is that when people disagree with others, they will want to speak more. In this context, speaking more may be an effort to delay an undesired decision or to gradually sway other Panelists in a direction that the speaker considers more appropriate. Speaking more may also be an effort to present a range of different arguments in support of the speaker’s position, in an effort to find some arguments that appeal to others and may help change their minds. Finally, as the speaker continues to explain their reasoning, they may expect to garner some other allies within the Panel who may, in turn, help convince yet other Panelists. Since most Panel recommendation are in favor of device approval, a talkative Panelist who is not in favor may have to be exceptionally convincing to affect their colleagues enough to alter such a recommendation.

On the other hand, women were nominally but not significantly less likely to abstain or vote against approval than men, and they did speak significantly less than men. That women spoke less is consistent with prior studies that show that women are less likely to participate in deliberations and decisions when they are the minority in a group, as they often are on FDA Panels [11]. Indeed, women accounted for only 22% of Panel members during the period studied. In addition, even women highly skilled at problem solving have been shown to be reluctant to engage in complex analyses when these occur during a process that is perceived as competitive [12]. To the extent that during a Panel meeting Panelists may be vying to speak and convince colleagues when time for both is limited, this part of Panel deliberations may be perceived as competitive and dissuade more women from speaking. Needless to say, receiving reduced input per Panelist from the already smaller cohort of women on Panels is not desirable, and this problem may be mitigated by applying current understanding of group dynamics. Specifically, majority female Panels could be constituted and the process of being recognized to speak by the chair made less competitive, with each Panelist given a fixed amount of time to speak and ask questions.

Biostatisticians appear to play a key role on Panels. They spoke significantly more than physicians during meetings, at least in part because biostatisticians were often asked questions by other Panelists regarding interpretation of pivotal trial results and appropriateness of analytic methods used. Further, meetings in which voters were able to reach unanimity with regard to final approval recommendation were comprised of a greater proportion of biostatisticians compared to those which were not unanimous.

In general it appears that biostatisticians are critical for interpreting the often voluminous and complex datasets that Panelists are asked to evaluate during the course of Panel meetings. FDA and the corporate sponsors do separately present detailed evidence regarding safety and effectiveness for the Panelists’ consideration, but many clinically adept Panelists may still struggle to understand the details of specific analyses, to what extent the data are definitive, and whether study design and data analysis were adequate. As members of the Panel itself, biostatisticians are perceived as relatively unbiased by other Panel members, and biostatisticians are also deemed to be expert at judging the integrity of the process whereby quantitative data was collected and assessed. To the extent that biostatisticians can reassure other Panelists that the data collection was precise, data analysis was performed correctly, and differences between experimental and control groups were valid, then the Panelists can be confident that their judgments regarding the suitability of a drug or device for approval are built on a solid foundation. This increased certainty, in turn, allows Panelists to be willing to come to agreement and make collective decisions, as versus in the situation where a lack of confidence makes some Panelists reluctant to commit. When disagreements occur among Panelists who are clinicians, biostatisticians, by virtue of being perceived as agnostic about technical medical issues as well as inherently dispassionate and data-driven, may be able to cool overheated tempers and return the Panelists to the more neutral ground of checking the numbers and effect size. Non-clinician Panel members, such as those expert on the underlying technology of a device, may be less able to follow the nuance of discussion of clinical issues but may feel more comfortable with the statistical analyses, as explained by the biostatistician. In sum, having more biostatisticians on the Panel may improve, in a more uniform manner, the understanding of study design and data interpretation among voting members leading to greater voter consensus.

Non-physician, non-biostatistician voters, on the other hand, appear to be unfamiliar with, or unwilling to participate in, the consensus process that is integral to a Panel meeting. Possibly their frame of reference is different than that of physician and biostatistician voters, and they may be expecting a higher level of scientific evidence than is available in human clinical trials.

Almost a quarter of voters were so-called uncertain voters, who asserted either that the device under review was effective but not safe, or safe but not effective. While a majority of uncertain voters cast final votes against approval, a substantial minority voted to approve. When they did cast a vote in favor of approval, uncertain voters appeared to place more weight on device safety than effectiveness, presumably because they felt uncertain effectiveness was less potentially harmful to patients than uncertain safety. The odd behavior of uncertain voters may be ascribed to a high level of doubt about the right course of action.

Although only 69% of individual votes were in favor or recommending device approval, 88% of Panels ultimately endorsed device approval. This appears related to the voting mechanism, whereby one or a few voting member’s strong opposition cannot override a majority vote in favor of approval.

The words spoken during the Panel deliberation session immediately preceding the final approval vote accounted for, on average, over three-quarters of the total words spoken by voting members during the meeting. This suggests that voting members typically wait to express their opinions and arrive at judgments, doing more listening than talking during earlier meeting segments.

That voting members seldom ask questions during OPH Q&A sessions, and if they did directed these to physician speakers or society representatives, not patients, is consistent with a recent survey study of MDAC Panel members. Survey respondents reported that OPH sessions were generally not influential in their recommendation decision, but they did find comments from society representatives to be helpful (M. Alam, A. Maisel, B. Cressey, et al.; unpublished data; December 2019).

Experienced voters may be more willing to vote against approval because, by attending more meetings, they have gained experience reviewing and evaluating evidence that provides them increased confidence in their assessments. Conversely, less experienced members may be less confident in their ability to properly sift the data and hence reluctant to vote against a majority. It appears that there is a significant learning curve for Panelists, with more experienced Panelists better able to perform the critical function of illuminating shortcomings in devices.

While we were limited by the number of Panel meetings that occurred in the time period of interest, the decision to restrict the inclusion criteria to a relatively short, recent time period was intentional. Since the rules governing functioning of FDAC Panel meetings has evolved over time (e.g. types of devices being approved, conflict of interest rules, gender balance, etc.), a longer time window would have resulted in internal validity issues, and been less generalizable to current Panels. Future studies may assess whether the current findings are generalizable to different contexts, such as FDA drug (CDRH) Panels convened for premarket approval. Future studies may also include content analysis of the words being spoken during Panel meetings.

Overall, this analysis is reassuring in that it shows that MDAC Panel members feel comfortable disagreeing with the Panel majority when they are concerned about some aspect of the device under review. In general, Panel members appear to weigh safety more than effectiveness, as is reasonable given the potential harm that can occur from approving unsafe products. While most members vote in favor of approval, those with greater Panel experience, uncertainty in their opinions, and professions other than medicine or biostatistics are more likely to vote against approval, and their inclusion is likely useful for ensuring Panels have the opportunity to hear and consider opposing views. Biostatisticians play an important role on Panels, helping to inform voters regarding technical aspects of data analysis, and bringing them to consensus.

## References

[1] Smith JF, Townsend SA, Singh N, Ma P. FDA advisory committee meeting outcomes. Nat Rev Drug Discov. 2012;11(7):513–4. doi: 10.1038/nrd3747 22743972

[2] Center for Devices, Radiological Health. Committee vacancies on CDRH. U.S. Food and Drug Administration. Available from: https://www.fda.gov/advisory-committees/advisory-committee-vacancies-qualifications-and-experience/medical-devices-and-radiation-emitting-products-committee-vacancies. Last accessed December 16, 2021.

[3] Guidance for FDA Advisory Committee Members and FDA Staff: Voting Procedures for Advisory Committee Meetings. Rockville MD: US Department of Health and Human Services, Food and Drug Administration; 2008. Available from: https://www.fda.gov/media/75426/download

[4] Rettig RA, Earley LE, Merrill RA, eds. Committee Operations. In: Food and Drug Administration Advisory Committees. Washington, DC: The National Academies Press; 1992:173–95. 25144034

[5] Steinbrook R. Financial Conflicts of Interest and the Food and Drug Administration’s Advisory Committees. N Engl J Med. 2005;353(2):116–8. doi: 10.1056/NEJMp058108 16014880

[6] Lurie P, Almeida CM, Stine N, Stine AR, Wolfe SM. Financial Conflict of Interest Disclosure and Voting Patterns at Food and Drug Administration Drug Advisory Committee Meetings. JAMA. 2006;295(16):1921–8. doi: 10.1001/jama.295.16.1921 16639051

[7] Broniatowski DA, Magee CL. Does Seating Location Impact Voting Behavior on Food and Drug Administration Advisory Committees? Am J Ther. 2013;20(5):502–6. doi: 10.1097/MJT.0b013e31821109d5 21642834

[8] United States Food and Drug Administration. Medical Devices Advisory Committee. Available from: https://www.fda.gov/AdvisoryCommittees/CommitteesMeetingMaterials/MedicalDevices/MedicalDevicesAdvisoryCommittee/default.htm. Last accessed August 10, 2020.

[9] Wardle M, Cederbaum K, & de Wit H. Quantifying talk: developing reliable measures of verbal productivity. Behavior research methods. 2011;43(1), 168–178. doi: 10.3758/s13428-010-0019-y 21287128 PMC3049967

[10] MacLaren NG, Yammarino FJ, Dionne SD, Sayama H, Mumford MD, Connelly S, et al. Testing the babble hypothesis: Speaking time predicts leader emergence in small groups. The Leadership Quarterly. 2020;31(5):101409.

[11] Karpowitz C. Mendelberg T., Shaker L. Gender Inequality in Deliberative Participation. American Political Science Review. 2012;106(3), 533–547.

[12] Niederle M. and Vesterlund L. Do Women Shy Away From Competition? Do Men Compete Too Much? Quarterly Journal of Economics. 2007;122(3).

